# The role of post-transcriptional regulation in SARS-CoV-2 infection and pathogenicity

**DOI:** 10.3389/fimmu.2023.1256574

**Published:** 2023-11-15

**Authors:** Xuan Wang, Zecheng Chang, Tingting Zhao, Weiyao Zhong, Jingwei Shi, Guoqing Wang, Xuesong Xu

**Affiliations:** Key Laboratory of Pathobiology Ministry of Education, College of Basic Medical Sciences/China-Japan Union Hospital of Jilin University, Jilin University, Changchun, China

**Keywords:** post-transcriptional modification, SARS-CoV-2, pathogenicity, virus-host interaction, regulation

## Abstract

The COVID-19 pandemic caused by the Severe Acute Respiratory Syndrome Coronavirus 2 (SARS-CoV-2) virus has had a significant impact on global social and economic stability. To combat this, researchers have turned to omics approaches, particularly epitranscriptomics, to limit infection and develop effective therapeutic strategies. Multi-omics can provide the host response dynamics during multiple disease phases to reveal the molecular and cellular landscapes. Epitranscriptomics focuses on the mechanisms of gene transcription in cells and tissues and the relationship between genetic material and epigenetic regulation. This review highlights the role of post-transcriptional regulation in SARS-CoV-2, which affect various processes such as virus infection, replication, immunogenicity, and pathogenicity. The review also explains the formation mechanism of post-transcriptional modifications and how they can be regulated to combat viral infection and pathogenicity.

## 1 Introduction

**Figure 1:**
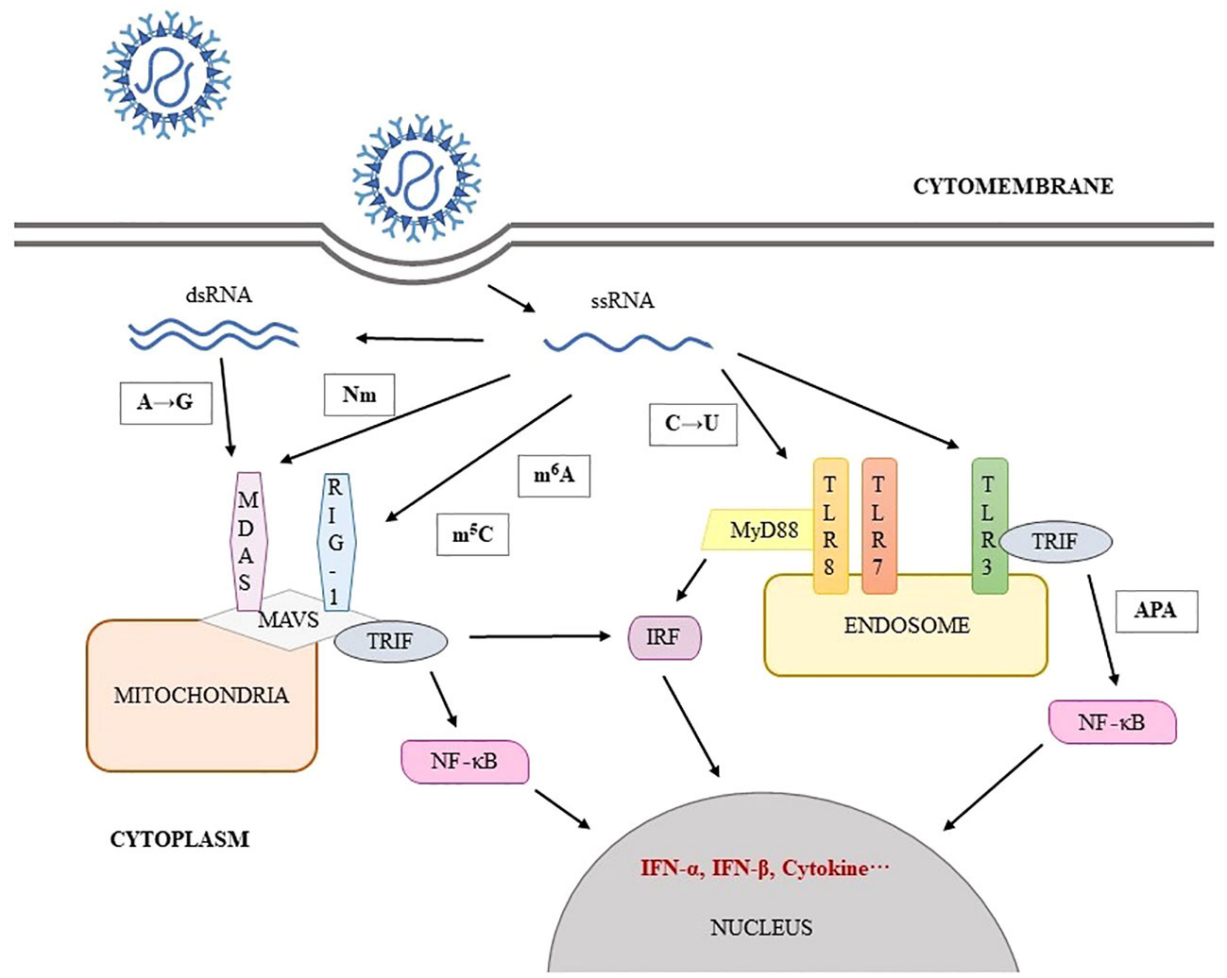
SARS-CoV-2 RNA modification involve in immune escape.

SARS-CoV-2 is an enveloped single-stranded positive-sense RNA virus with a genome size of approximately 29,903 nucleotides (1). The genome structure is sequentially 5’ UTR, ORF1a/b, spike (S)protein (21538~25359), envelope(E) protein (26220~26447), membrane(M) protein (26498~27166), nucleocapsid(N) protein (28249~29508), 3’ UTR and poly (a) tail. Following the entry of SARS-CoV-2, the full-length negative-sense template is synthesized from the positive-sense genomic RNA (gRNA) and made as a template for progeny viral RNA synthesis. Subgenomic negative-sense templates are also synthesized from discontinuous transcription and serve as templates for mRNA synthesis (2). The gRNA is packaged by the structural proteins to assemble progeny virions. Shorter subgenomic RNA (sgRNA), RNA that results from virus replication of partial genomic regions which process may involve specific internal transcriptional initiation and termination sites, encode conserved structural proteins and several accessory proteins (3). To investigate the functional aspects of viral proteins, replication mechanism, and host-viral interactions involved in pathogenicity, it is necessary to have a clear understanding of the expressed sgRNA and ORFs (3). Post-transcriptional modification is a common occurrence in tRNA, rRNA and mRNA of eukaryotes and prokaryotes. These modifications not only affect RNA self-stability but also its interaction with molecules related to physiological functions such as metabolism, carcinogenesis, and immunity (4). It’s shown that modified RNA will inhibit or evade innate immune stimulus (5). The modification of viruses through post-transcriptional processes has been noted since the 1970s (6). With the advancements in epitranscriptomics, the covalent modification of single nucleotides in mRNA has become an area of interest. For example, during herpes simplex virus-1 (HSV-1) infection, increasing methylation level of host mRNA and nuclear export result in a stronger antiviral response (7). Alternative splicing of mRNA is associated with activation of the noncanonical NF-κB pathway and this pathway is co-opted by Rift Valley fever virus to enhance viral success during infection (8). The published articles provide referential ideas and methods for SARS-CoV-2, there is similarity or difference compared to other virus. Currently, the map of SARS-CoV-2 post-transcriptional modification is being developed, with at least 41 modification sites on viral transcripts identified using nanopore Direct RNA Sequencing (DRS). The most frequent motif identified is AAGAA, which is enriched in genomic positions 28,500–29,500. Additionally, there is evidence of a modification mechanism specific to certain RNA species (3). Post-transcriptional modifications such as N^6^-methyladenosine(m^6^A), 2’-O-methylation (Nm), RNA editing, N^5^-methylcytosine(m^5^C), pseudouridine, alternative splicing and alternative polyadenylation (**Table 1**), have been found to be associated with the viral life cycle and anti-viral immune mechanism (**Figure 1**). SARS-CoV-2 enters human cells and causes COVID-19, with respiratory failure due to acute respiratory distress syndrome (ARDS) being the primary cause of death. Cytokine storms can lead to the destruction of host cells, resulting in multiple organ failure or death (41). In this review, we examine the impact of post-transcriptional regulation on virus infection and pathogenicity when SARS-CoV-2 infects host cells.

**Table 1:** Types of post-transcriptional modifications relevant to SARS-CoV-2.

Modification Types	Modification Sites	Identification Methods	Functions	References
N^6^-methyladenosine	DRm^6^ACH consensus motif	meRIP-seq, miCLIP	stability, decay, transport, and translation efficiency of SARS-CoV-2	(2, 9–13)
2’-O-methylation	2’-O sites of the 5’ cap structure	RNA-seq, structural studies (Cryo-EM, etc.)	RNA splicing, transport and protein synthesis	(14–21)
RNA editing	dsRNA, (+) RNA	RNA-seq	spread, replication, immunogenicity and pathogenicity of SARS-CoV-2	(22–31)
N^5^-methylcytosine	close to translation initiation codons	DRS	replication, host innate immunity of SARS-CoV-2	(3, 32–34)
Pseudouridine	TRS-S, TRS-3a, TRS-E, TRS-M	DRS	activate immune response	(35)
Alternative splicing	mRNA	RNA-seq, proteomic, interactomic	facilitate viral infection and replication, suppress the host innate immune system	(36–39)
Alternative polyadenylation	3’ UTR	RNA-seq	RNAPII transcriptional termination, mRNA stability, and translation efficiency	(40)

(+)RNA means positive-sense RNA.

**Figure 2:**
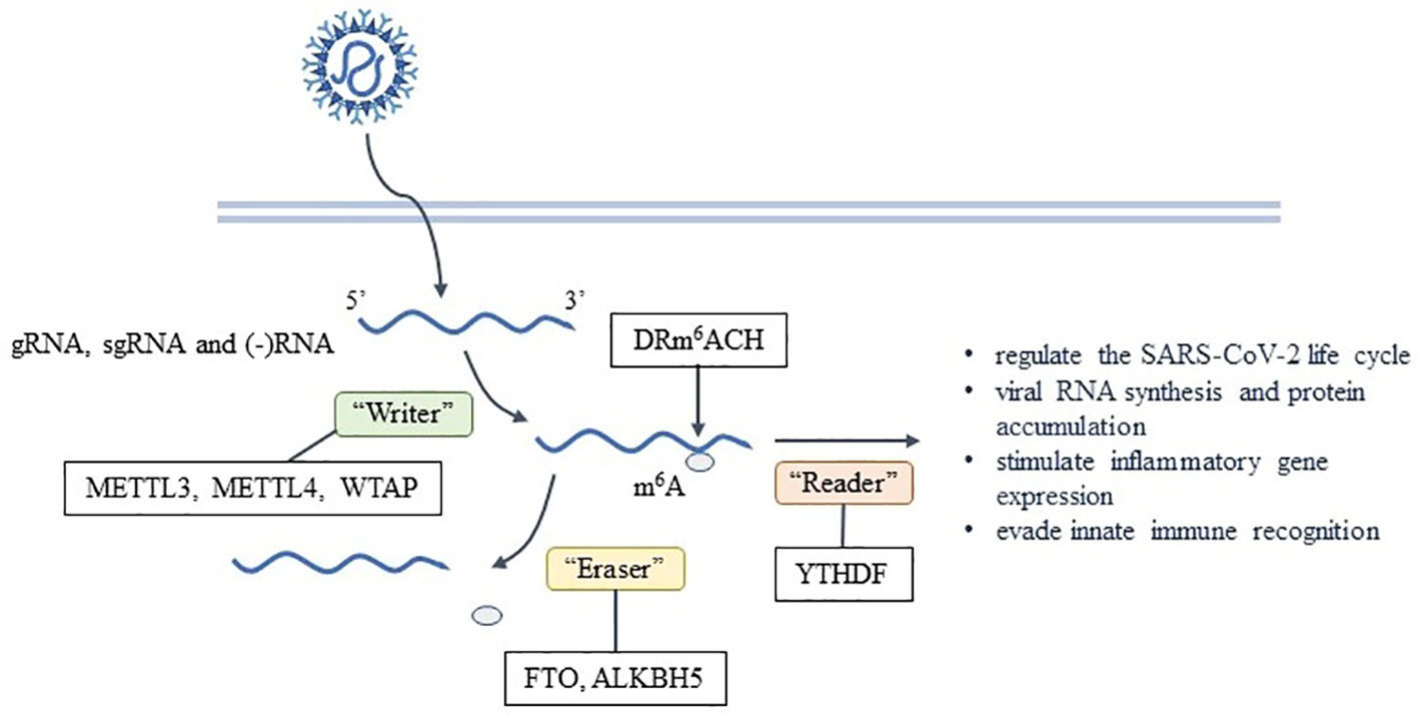
Formation and effects of m^6^A on SARS-CoV-2.

## 2 Types of post-transcriptional regulation

### 2.1 N^6^-methyladenosine(m^6^A)

m^6^A is the most common post-transcriptional modification found in eukaryotes and has been extensively studied. It’s also found in various viruses, including SARS-CoV-2 (9–11). The m^6^A modification on mRNA is usually located in the DRm^6^ACH consensus motif, where D represents A, G or U; R represents G or A; and H represents A, C, or U (2). Using the methylated RNA immunoprecipitation sequencing(meRIP-seq) technique, it has been determined that gRNA, sgRNA and negative RNA intermediates of SARS-CoV-2 all contain m^6^A (2, 12), which is enriched in the nucleocapsid (N) region of the viral genome (13). Currently, m^6^A individual-nucleotide-resolution cross-linking and immunoprecipitation (miCLIP) combined with meRIP-seq is used to report 8 high-confidence m^6^A sites (2). meRIP-seq can provide a qualitative analysis of hypermethylation mRNA regions but only in the genomic level. miCLIP can map m^6^A locations transcriptome-wide with single-nucleotide resolution, but cDNA library preparation uses radioactive labeling.

The catalysis of m^6^A is done by the methyltransferase complex known as the “Writer”, including the complex of methyltransferase-like protein 3 (METTL3), METTL14, and Wilms’ tumor 1-associating protein (WTAP), while the removal of m^6^A is done by the demethylase or “Eraser”, such as fat mass- and obesity-associated protein (FTO) and ALKBH5. The binding proteins or “Reader”, such as YTH domain family proteins(YTHDF), recognize and functionally interact with m^6^A (42). This process is dynamically reversible and regulated by enzymes (43). RNA interference experiments have shown that the depletion levels of “Writer” and “Reader” are negatively correlated with the replication and infection rate of SARS-CoV-2 (12). It has been observed that no virus has the capability to encode the “Writer” (4). The *METTL3* gene knockout has been found to reduce the level of m^6^A in SARS-CoV-2 (13). Hence, the host “Writer” is responsible for the formation of m^6^A after SARS-CoV-2 enters the cells (2). In SARS-CoV-2-infected cells, abundant METTL14 and ALKBH5 are repositioned into the cytoplasm to participate in the viral life cycle. The catalytic activity of METTL3 is necessary for efficient viral RNA synthesis and protein accumulation within 24 hours of infection (12). Inhibiting METTL3-mediated m^6^A leads to a reduction in viral load and pro-viral gene expression in host cells (13), as well as a decrease in N protein expression and the efficiency of gRNA and sgRNA synthesis (12). This ultimately affects the replication efficiency of SARS-CoV-2, resulting in a downregulation of virus production and transmission (12). Additionally, the YTHDF1 recognizes m^6^A-modified transcripts directly and promotes their translation by binding to eukaryotic translation initiation factors such as eIF3 (44). YTHDF2 leads to the decay of m^6^A-labeled transcriptomes, playing a major role in RNA degradation (2, 42), which is negative for the replication of SARS-CoV-2. “Writer” and “Reader” play varied roles in infection by different viruses, acting both directly on viral transcripts and indirectly to shape the innate immune response. RNA viruses are both negatively and positively regulated by m^6^A in context-specific manners, similar to context-specific effects of m^6^A and its regulatory proteins that have been reported for cellular mRNAs outside the context of infection (45).

All of above indicate that m^6^A is crucial to the life cycle of SARS-CoV-2. A properly functioning m^6^A modification pathway is essential for the reproduction of SARS-CoV-2, although the specific mechanism by which it influences viral replication and translation is not yet clear. In addition, some scholars claim that m^6^A negatively regulates the life cycle of SARS-CoV-2 by inhibiting its replication (2). However, the exact role of m^6^A in regulating SARS-CoV-2 infection and pathogenicity requires further investigation.

In addition to directly impacting SARS-CoV-2, m^6^A also affects the stability, decay, transport, and translation efficiency of the virus. Furthermore, it is possible that m^6^A may be utilized by the virus to evade pattern recognition receptor (PRR) detection or to be bound by precursor RNA binding proteins (4). After infection, immune response genes were highly enriched in *METTL3*-deficient cells, suggesting that the early response of host cells against viral infection may be directly related to the level of m^6^A. In fact, the up-regulation of chemokine/cytokine genes expression (including IL-8, CXCL1, CXCL3, and CCL20) in *METTL3*-deficient cells was dependent on viral infection rather than on induction of IFN gene (13). Moreover, virus binding with RIG-I during infection may play an important role in the expression of downstream inflammatory genes. Currently, it has been confirmed that reducing the m^6^A on SARS-CoV-2 can improve the recognition rate of RIG-I and enhance the induction of inflammatory genes (13). These results suggest that m^6^A is a strategy for SARS-CoV-2 to evade innate immunity (**Figure 2**).

### 2.2 2’-O-methylation (Nm)

The cap structure at the 5’ end of viral mRNAs plays essential roles in the life cycle of a virus by promoting initiation of translation, protecting mRNAs, and helping the virus escape host immune recognition (14). mRNA in most eukaryotic cells and virus are methylated at the N-7 and 2’-O sites of the 5’ cap structure, catalyzed by methyltransferase in the nucleus or cytoplasm to form m^7^GpppN (Cap-0) and m7GpppNm (Cap-1 or Cap-2) (46). A large number of studies have shown that methylation of cap structure controls gene expression by regulating RNA splicing, transport and protein synthesis (15). Nm is necessary for the normal life cycle of the virus (16, 43). Current studies suggest that 2’-O-methylation may be a means by which hosts differentiate between their own and non-own RNA (46). Many RNA virus that cannot enter the host nucleus (including flaviviruses, coronaviruses, poxviruses, paramyxoviruses, etc.) fail to use the classical capping mechanism of the host, so they gradually evolve their own enzymes to catalyze the formation of cap-like structures similar to host mRNA, which are translated in the host organelles as Cap-0 structures and then escape the recognition of the immune system by Nm (43). Beyond inducing IFN-β expression in an MDA5-dependent manner, the absence of Nm triggers another antiviral mechanism, namely IFIT-1-mediated viral replication restriction (16). IFIT1, an RNA-binding protein induced by IFN, competitively binds 5’mRNA with translation initiation factor eIF4E. However, IFIT-1 preferentially binds to Nm-lacking viral RNA and inhibits its translation due to higher affinity (43).

**Figure 3:**
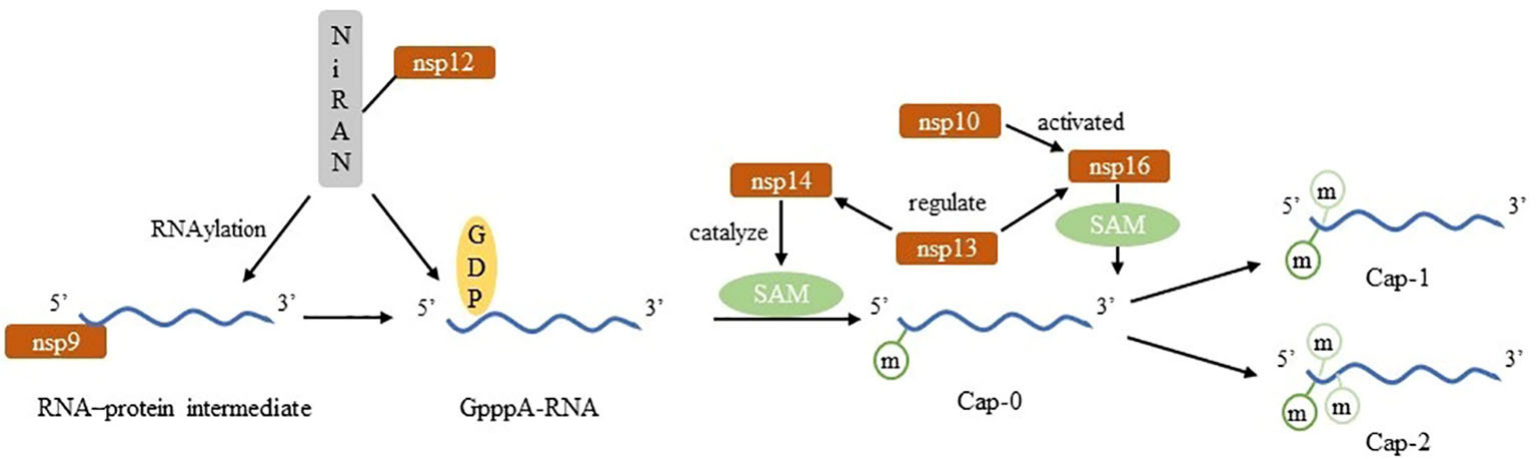
Construct of Nm in SARS-CoV-2.

SARS-CoV-2 nsp14 acts as N-7-methyltransferase and nsp16 does 2’-O-methyltransferase. nsp14 modified neonatal viral transcripts in a non-sequence-specific manner to generate Cap-0 structure while nsp16 is a m^7^GpppA-specific, S-adenosylmethionine (SAM) -dependent 2’-O-methyltransferase that binds to nsp10 and is activated to catalyze the binding of RNA substrates to methyl donors. SAM, a universal methyl group donor, SAM-dependent methyltransferases facilitate the transfer of a methyl group to a variety of substrates (47).That is unique to SARS-CoV-2. Besides, nsp13 plays a role in regulating the activities of those two transferases (15, 17). The kinase-like nidovirus RdRp-associated nucleotidyltransferase (NiRAN) domain of nsp12 transfers the RNA to the amino terminus of nsp9, forming a covalent RNA–protein intermediate (a process termed RNAylation). Subsequently, the NiRAN domain transfers the RNA to GDP, forming the core cap structure GpppA-RNA. The nsp14 and nsp16 methyltransferases then add methyl groups to form functional cap structures. The nsp13 protein produces GDP from GTP, which binds to the NiRAN active site and attacks RNAylated nsp9, releasing capped RNA and regenerating unmodified nsp9 (18). The progress of Nm relevant to SARS-CoV-2 is shown in **Figure 3**.

Loss of 2’-O-methylase activity results in significantly reduction of SARS-CoV-2 infection, which is characterized by decreased viral replication and limited dyspnea in animal models (19). Disruption of SARS-CoV-2 nsp16 function attenuates viral replication in a type I interferon-dependent manner (20). If SARS-CoV-2 lacks Nm, MDA5 will recognize its RNA and be activated to interacts with mitochondrial antiviral signaling proteins (MAVS), which phosphorylates IRF3 and IRF7 to transcript, synthesize and secret of IFN-α and IFN-β. IFNs bind to their receptors to initiate signaling cascades that induce the expression of multiple cytokines, establishing the antiviral state (21, 43). In addition, RIG-I specifically recognizes 5’-ppp on ssRNA and part of dsRNA. RIG-I activity will be downregulated when 5’-ppp contains modified nucleotides (16). It proves that Nm of viral mRNA is a mechanism by which virus could evade recognition of non-indigenous RNA by the host innate immune system. Therefore, Nm has a positive effect on the replication and pathogenicity of SARS-CoV-2.

### 2.3 RNA editing

RNA editing is a widespread nucleotide modification mechanism. Compared with dsRNA virus, ssRNA virus may be more prone to base alkylation or chemical deamination (22). This class of RNA editing events is catalyzed by APOBEC and ADAR. Although RNA editing essentially occurs in endogenous mRNA of cellular organisms regardless of infection (48). But when RNA virus invade cells, edit-related enzymes will act as a defense system to limit viral activity (49), meaning that the RNA editing events that occur in the viral genome are a kind of host self-preservation mechanism. However, it is still possible that viral RNA editing events originate from mechanisms other than ADAR and APOBEC such as RNA transcription and reactive oxidative species. A major source of mutations in viruses and other organisms are transcription errors by cellular RNA or DNA polymerases that are incorporated during replication. Different types of polymerases may show varied mutational profiles with separate propensities to mis-incorporate particular transitions or transversions. It has been confirmed that SARS-CoV-2 undergoes ADAR-mediated A→I and APOBEC-mediated C→U RNA editing in human cells (23–25). Additionally, it has been recently proposed that substitutions occurring in the SARS-CoV-2 genome, notably the G→U transversion may originate from mutational damage to viral RNA during periods of oxidative stress (26). A→G effectively limits virus transmission and thus reduces the number of viral progeny expressing this modification. In contrast, C→U remains in viral progeny and can be repaired during viral adaptation (24, 27).

**Figure 4:**
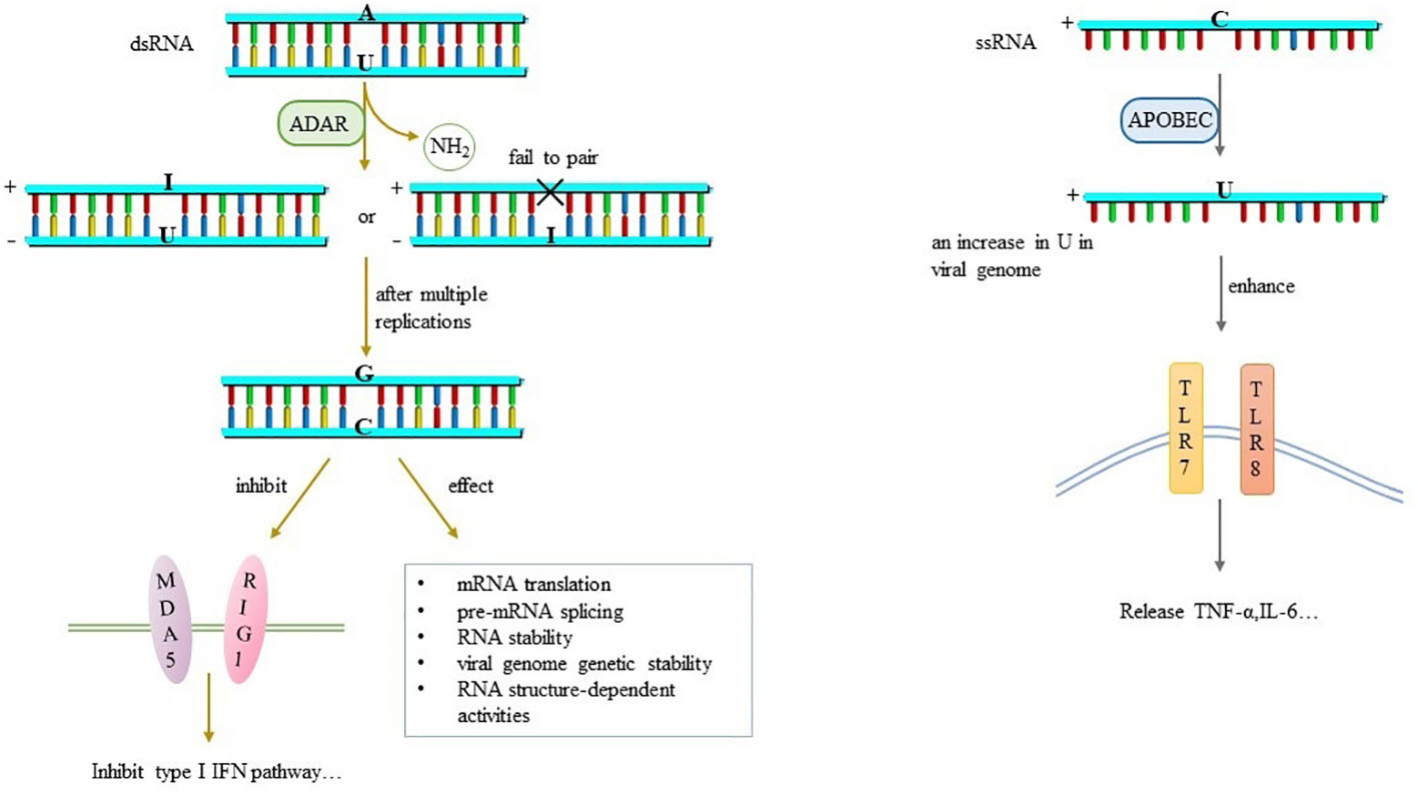
RNA editing and its effects.

A→I is a dynamically regulated modification event related to the strength of congenital immune response, and is more commonly seen in samples with low viral load (25). A→I relies on ADAR deamination and is fixed as A→G after multiple replications (24). The negative strand RNA with A→G cannot be used as a template for the synthesis of positive strand RNA, so it loses the ability to reprogram or regulate the viral genome, which affects the stability and replication efficiency of RNA (27). It varies among different tissues and cell types. A-to-I editing may effect gene expression and function in virus-infected cells by a number of ways that includes: mRNA translation, by changing codons and hence the amino acid sequence of synthesized proteins; pre-mRNA splicing, by changing a conserved A in splice site recognition sequences; RNA stability by altering sequences and structures involved in nuclease recognition; viral genome genetic stability, by changing template and thus product sequences by deamination leading to A-to-G (U-to-C) transitions during viral RNA synthesis; and RNA structure-dependent activities including microRNA production or targeting or dsRNA protein-RNA interactions involved in innate immune responses (50). For immunology, dsRNA containing multiple groups of I/U inhibits the activity of MDA5, RIG1 and IRF-3, thus inhibiting the induction pathway of IFN-I and affecting the host antiviral response (5, 28) (**Figure 4**).

A→I, which mainly occurs in dsRNA products of SARS-CoV-2, including positive and negative strand RNA, is associated with the spread, replication, immunogenicity and pathogenicity of SARS-CoV-2 (24). Leukocyte whole-genome RNA sequencing data find reduced A-to-I editing of Alu dsRNA in patients with severe COVID-19 disease. dsRNA of unedited Alu is an effective activator of downstream IRF and NF-κB driven transcriptional response, which may lead to increased inflammatory response and is related to disease severity (29). In addition, A→G editing sites are found in the S protein of SARS-CoV-2, which either changes the immunogenicity of S protein epitopes, or enhances RBD expression and ACE2 affinity, affecting viral characteristics such as infectivity, pathogenicity and host range in various ways (25, 27). Nevertheless, the overall expression level of A→G in SARS-CoV-2 is still low and has little effect on viral genome (27).

C→U is catalyzed by APOBEC and biased towards positive strand RNA, which occurs late in the virus life cycle (24). Previously, many studies have shown that U-rich ssRNA stimulates innate immune cells to produce inflammatory cytokines through TLR7 signal transduction (30) (**Figure 4**). Analysis of SARS-CoV-2 genome mutations database shows a very high mutation rate, accounting for about 40% of all single nucleotide variants, suggesting that host APOBEC is involved in RNA editing mechanisms except its own mutations (28). It speculates that the reduction of SARS-CoV-2 cytosine is an escape strategy to negate the antiviral effect of APOBEC (31). Later experiments have confirmed it that C→U editing in SARS-CoV-2 genome leads to an increase in U in viral genome, which enhances the activation of TLR7 and TLR8. Further, more pro-inflammatory cytokines such as TNF-α and IL-6 are produced (30)to affect the pathogenicity and immunogenicity of the virus, but the specific mechanism needs to be further explored.

For SARS-CoV-2, nucleotide changes caused by RNA editing may be a direct source of genetic variation, influencing the evolution and plasticity of the virus. Since most of their genome encode proteins directly, this modification is likely to lead to changes in amino acids that further modify protein products (23).

### 2.4 N^5^-methylcytosine(m^5^C)

In recent years, it has found that another modification, N^5^-methylcytosine. The primary writers for m^5^C methylation of mRNA in animals have been proposed to be NSUN2 and TRDMT1 (DNMT2). It was reported that ALYREF and YBX1 served as potential m^5^C readers that could recognize m^5^C-modified mRNA and mediate mRNA export from the nucleus or affect the stability of their target mRNAs. Nevertheless, the demethylases responsible for removing m^5^C methylation on mRNA have not been clearly identified (32). It has been reported that m^5^C sites detected by DRS technology in the absence of negative control are likely to be false positive. It has been proposed that m^5^C residues, which are often located close to translation initiation codons, can promote mRNA translation and enhance nuclear RNA export (33). Currently, m^5^C has been shown to be an active regulator of viral replication (34), which exerts various transcriptional regulatory activities in different modification sites (51). m^5^C appears to enhance the binding affinity of viral dsRNA to RIG-I, but fails to induce the conformational changes to activate the antiviral signaling cascade (5), thus affecting the host innate immunity. m^5^C also exists in SARS-CoV-2 (3). Moreover, NSUN2, a typical m^5^C methyltransferase, negatively regulates type I interferon responses during various viral infections, including SARS-CoV-2. It serves as a negative regulator of interferon response by accelerating the fast turnover of IRF3 mRNA, while endogenous NSUN2 levels decrease during SARS-CoV-2 and various viral infections to boost antiviral responses for effective elimination of viruses (32).

### 2.5 Alternative splicing

AS is a basic mechanism to regulate proteome diversity by splicing single RNA to produce alternative mRNAs that encode different protein subtypes in structure and function (52). Recent advances in RNA sequencing technologies revealed that up to several hundreds of host genes can show altered mRNA splicing upon viral infection. Viruses that gain access to the splicing machinery have evolved an expansion of their coding capacity by producing spliced viral mRNAs. The observed changes in AS events can be either a direct consequence of viral manipulation of the host splicing machinery or result indirectly from the virus-induced innate immune response or cellular damage. It is essential for cell cycle, nuclear transport of stress response and immune response (53). Some results suggest that host splicing machinery is profoundly dysregulated owing to SARS-CoV-2 infection, which could affect host immune response by altering AS of cellular genes (36). The most common local splicing in SARS-CoV2 infection is exon jumps (ES, 41.8%), followed by complex events (36.6%), intron retention (10.3%), alternative 3’ splicing sites (5.9%) and alternative 5’ splicing sites (5.4%). Virus-induced changes in transcript subtypes are widespread in PBMC samples from COVID-19 patients and are closely related to the severity of the disease process (37). nsp16 binds to the mRNA recognition domains of the U1 and U2 components of the spliceosome and inhibits global mRNA splicing in the cells of patients, thereby reducing the innate immune response of host cells to virus recognition (38). Proteomic analysis of the virus-host protein interaction network revealed that 13 splicing factors are down-regulated in COVID-19 patients, including DEAD-box 5(DDX5), DDX17 and DDX1, indicating that SARS-CoV-2 infection result in serious dysfunction of host splicing mechanism (37). DDX RNA helicases play essential roles in a broad array of biological processes and serve multiple roles at the virus-host interface. For example, DDX3X has already been validated as a target for broad-spectrum antiviral molecules against a number of RNA viruses. DDX5 plays fundamental roles in transcriptional regulation and in viral replication (54). In addition, host RNA helicases may be involved in SARS-CoV-2 replication. It has demonstrated that DDX21 and MOV10 RNA helicases limit viral infection and replication. SARS-CoV-2 seems to hijack host cellular RNA helicases to play a proviral role by facilitating viral infection and replication and by suppressing the host innate immune system (39).

### 2.6 Pseudouridine(ψ)

ψ is formed by the isomerization of U catalyzed by pseudouridine synthases (PUS) and has been shown to locate at diverse sites, accumulating in tRNA and rRNA but remaining in low quantities in mRNA. Using DRS, five high confidence ψ sites in transcription-regulatory sequence-S(TRS-S), five in TRS-3a, five in TRS-E, and five in TRS-M of SARS-CoV-2 sgRNAs were detected (35).ψ may be associated with AS during β-coronavirus infection (38). ψ instead of uracil will attenuate mRNAs to activation of immune response but retain its protein-coding ability. This conclusion has been applied to design a class of mRNA vaccines. There are few literatures reporting the direct association between SARS-CoV-2 infection and ψ.

### 2.7 Alternative polyadenylation

APA is a gene regulatory mechanism that produces different 3’ terminus and influences many aspects of mRNA metabolism, including RNAPII transcriptional termination, mRNA stability, and translation efficiency. The shortening of 3’UTR caused by APA affects not only the number of miRNA-binding sites but also the targeting efficiency of miRNA (55). Studies prove that APA may participate in antiviral innate immune response by regulating mRNA abundance, protein expression and influencing viral replication, and may regulate signal transduction, which increases the biological complexity of antiviral response (56). Dynamic APA events were identified in RNA-Seq data from PBMC of COVID-19 patients. Most of the events showed a short 3’ UTR. The standard poly (A) signal, AATAAA, was successfully identified by HOMER motif enrichment analysis in dynamic APA sites. APA is further found to be involved in IFN signaling in the antiviral innate immune response of COVID-19 patients. Besides, gene expression of APA and its regulatory factors are interfered in virus-infected cells. APA is associated with expression levels of 3’ UTR processing factors in COVID-19 patients (40).

### 2.8 Other post-transcriptional modifications

In addition to the mentioned types, other post-transcriptional modification processes of SARS-CoV-2, such as N^1^-methyladenosine (m^1^A) and N^3^-methylcytosine (m^3^C), have also been reported in some papers (57), but few of them have come into focus. The effects of those modification on viral infection and pathogenicity remain explored.

## 3 Summary and prospect

Since the outbreak of SARS-CoV-2 in 2019, numerous experts and scholars have conducted research and exploration in various fields, gradually unveiling the mystery of the virus. With the development of epitranscriptomics, the transcriptome structure and modification spectrum of SARS-CoV-2 have emerged. The influence of chemical modification formed in the process of viral replication, transcription and translation on its genome expression is closely related to viral infection and pathogenicity. By directly acting on the life cycle of the virus or indirectly regulating the immune response of the host against the invasion of the virus, modified viral RNA blaze a trail for their own transmission, pathogenesis and immune escape in the host-virus interaction. These mechanisms also provide strategies and ideas for the medical community to limit the virus, prevent and treat the disease. It is of practical significance to trace the origin and evolutionary direction of virus, find the way to prepare attenuated vaccine and develop new antiviral drugs.

Among the post-transcriptional modification that have been confirmed to exist in SARS-CoV-2, m^6^A, Nm, RNA editing, AS, and APA have been widely reported. The clear understanding of their formation mechanism, dynamic regulation, effects, and related enzymes and cofactors provides a reference for the majority of scholars and researchers. However, some other post-transcriptional modification processes, such as pseudouridine(ψ), are still lack of literature at present. So whether it really exists in SARS-CoV-2 and whether it can participate in the virus infection and the pathogenic process may need further attention and research. However, most experiments ignore the effect of viral modification on the secondary structure of RNA, which may become a problem to be considered in the future. In addition, with the spread and development of the SARS-CoV-2 variant, the epidemic occurred again in China at the end of 2022. A new round of thinking and discussion may be triggered by such questions as whether the modification characteristics and tendency of virus deviate from the previously revealed conclusions, whether the post-transcriptional process caused by the virus entering the organism is closely related to virulence, and virus-host interaction.

## Author contributions

XW: Visualization, Writing – original draft, Writing – review & editing. ZC: Writing – original draft, Writing – review & editing. TZ: Data curation, Writing – review & editing. WZ: Writing – original draft, Data curation. JS: Writing – original draft, Project administration. GW: Conceptualization, Funding acquisition, Methodology, Writing – review & editing. XX: Funding acquisition.
